# Protective Effects of Aqueous Extract of *Luehea divaricata* against Behavioral and Oxidative Changes Induced by 3-Nitropropionic Acid in Rats

**DOI:** 10.1155/2015/723431

**Published:** 2015-10-28

**Authors:** Aline Alves Courtes, Letícia Priscila Arantes, Rômulo Pillon Barcelos, Ingrid Kich da Silva, Aline Augusti Boligon, Margareth Linde Athayde, Robson Luiz Puntel, Félix Alexandre Antunes Soares

**Affiliations:** ^1^Universidade Federal do Pampa (UNIPAMPA), Campus Uruguaiana, Uruguaiana, RS, Brazil; ^2^Departamento de Bioquímica e Biologia Molecular, Centro de Ciências Naturais e Exatas (CCNE), Universidade Federal de Santa Maria (UFSM), Santa Maria, RS, Brazil

## Abstract

Huntington's disease (HD) is an autosomal dominant neurodegenerative disease. Accordingly, 3-nitropropionic acid (3-NP) has been found to effectively produce HD-like symptoms. *Luehea divaricata* (*L. divaricata*), popularly known in Brazil as “açoita-cavalo,” may act as a neuroprotective agent *in vitro* and *in vivo*. We evaluated the hypothesis that the aqueous extract of *L. divaricata* could prevent behavioral and oxidative alterations induced by 3-NP in rats. 25 adult Wistar male rats were divided into 5 groups: (1) control, (2) *L. divaricata* (1000 mg/kg), (3) 3-NP, (4) *L. divaricata* (500 mg/kg) + 3-NP, and (5) *L. divaricata* (1000 mg/kg) + 3-NP. Groups 2, 4, and 5 received *L. divaricata* via intragastric gavage daily for 10 days. Animals in groups 3, 4, and 5 received 20 mg/kg 3-NP daily from days 8–10. At day 10, parameters of locomotor activity and biochemical evaluations were performed. Indeed, rats treated with 3-NP showed decreased locomotor activity compared to controls. Additionally, 3-NP increased levels of reactive oxygen species and lipid peroxidation and decreased ratio of GSH/GSSG and acetylcholinesterase activity in cortex and/or striatum. Our results suggest that rats pretreated with *L. divaricata* prior to 3-NP treatment showed neuroprotective effects when compared to 3-NP treated controls, which may be due to its antioxidant properties.

## 1. Introduction

Huntington's disease (HD) is an autosomal dominant progressive neurodegenerative disorder, characterized by motor dysfunction, emotional disturbances, abnormal involuntary movements, dementia, and weight loss [1, 2]. The neuropathological changes associated with these physical symptoms of HD include progressive neuronal degeneration and atrophy primarily affecting the striatum and cerebral cortex [3, 4]. This neurodegenerative disorder is believed to be caused by an expanded trinucleotide CAG sequence in exon 1 of the Huntingtin gene (Htt), which encodes a stretch of glutamines in the Huntingtin protein [5]. Formation of Htt aggregates and alteration of overall gene expression profiles have also been reported in peripheral tissues of HD patients [6, 7]. Compelling evidence also exists that mutant Huntingtin alters mitochondrial trafficking and function [8, 9].

HD-like symptoms may be induced experimentally in animal models through the administration of specific neurotoxins. 3-Nitropropionic acid (3-NP), a natural neurotoxin produced by several species of fungi (*Aspergillus flavus *and* Astragalus arthrinium*) and plants (*Indigofera endecapylla*) [10, 11], has been used successfully to induce HD-like symptoms in experimental animals [12, 13]. The mechanism by which 3-NP induces neurotoxicity involves the irreversible inhibition of succinate dehydrogenase (SDH) [14, 15], which results in mitochondrial dysfunction, as evidenced by intracellular energy failure and oxidative stress [16, 17]. 3-NP-treated animals present with motor-behavioral disorders, including gait, an inability to balance over a narrow beam, deficits in foraging or exploratory behaviors and cognition, and increased anxiety and/or depression [15, 18]. Since it is generally recognized that 3-NP administration induces HD-like symptoms in animals with a phenotype similar to the inherited human disease, this model represents a valuable tool to evaluate the effect of novel therapies to treat HD [19].

Therapeutic strategies aimed at preventing or delaying neuronal degeneration represent a reasonable choice for the treatment of neurodegenerative diseases [4, 20, 21]. Accordingly, there is a growing interest in the use of natural antioxidants, including polyphenols found in medicinal and dietary plants that might prevent cell death and damage associated with the administration of various neurotoxins [13, 22–24].

The naturally occurring plant* Luehea divaricata* Mart. (Tiliaceae) (*L. divaricata*), popularly known in South America as “açoita-cavalo” [25, 26], contains numerous polyphenols. This plant has been used traditionally in folk medicine to treat dysentery, leucorrhea, rheumatism, blennorrhea, tumors, bronchitis, and skin lesions, among others [26–28]. A phytochemical screening of* L. divaricata* leaves has revealed the presence of flavonoids, tannins, saponins, and mucilage. Additionally, alkaloids, fixed oils, anthocyanidins, carotenoids, and polysaccharides have also been found to be present in crude extracts of* L. divaricata* [28]. Although aqueous herbal extracts have attracted recent attention since they can be consumed in a daily basis as a decoction, few studies have evaluated the potential neuroprotective therapeutic effects of aqueous extracts, prepared as a tea, from leaves of* L. divaricata*. Previous studies have reported genotoxicity of the aqueous extract of* L. divaricata* leaves [29], a cytostatic effect of the methanolic extract of the leaves and antimutagenic activity of the aqueous extract of the bark [30]. In addition to these previous reports, the design of our research studies was also based on (1) previous data supporting the rational search for therapeutic strategies that either potentiate endogenous antioxidants or reduce oxidative stress generation in order to delay HD progression and (2) the knowledge that infusion of the leaves of* L. divaricata* in hot water releases high concentrations of polyphenols and flavonoids [31, 32]. Given the growing interest in natural antioxidants, especially polyphenols, present in medicinal and food plants, the putative antioxidant properties of* L. divaricata* aqueous extracts, the involvement of oxidative stress in neurodegenerative disorders (HD-like symptoms) induced by 3-NP, and the paucity of evidence concerning the potential protective effect of* L. divaricata* in experimental models of neurotoxicity/neuropathology, we evaluated the hypothesis that pretreatment with the aqueous extract of* L. divaricata* could prevent or attenuate the neurobehavioral sequelae induced by 3-NP administration in rats. Using high performance liquid chromatography (HPLC), we also characterized the phytochemical profile of the* L. divaricata* extract used in our study.

## 2. Materials and Methods

### 2.1. Chemicals

3-Nitropropionic acid, thiobarbituric acid (TBA), malonaldehyde-bisdimethylacetal (MDA), and 2′,7′-dichlorofluorescein diacetate (DCFH-DA) were purchased from Sigma (St. Louis, MO, USA). All other reagents were obtained from local suppliers. Methanol, phosphoric acid, gallic acid, chlorogenic acid, caffeic acid, and rosmarinic acid were purchased from Merck (Darmstadt, Germany). Catechin, epicatechin, vitexin, rutin, quercetin, and luteolin were acquired from Sigma Chemical Co. (St. Louis, MO, USA). High performance liquid chromatography (HPLC-DAD) was performed with a Shimadzu Prominence Auto Sampler (SIL-20A) HPLC system (Shimadzu, Kyoto, Japan), equipped with Shimadzu LC-20AT reciprocating pumps connected to a DGU 20A5 degasser with a CBM 20A integrator, SPD-M20A diode array detector, and LC solution 1.22 SP1 software.

### 2.2. Plant Material

The leaves of* Luehea divaricata* Mart. (family Tiliaceae) were used as the plant material and were collected in Santa Maria (Rio Grande do Sul, Brazil). The collection of the leaves of* L. divaricata* was carried out during the flowering period, which occurs in December. The taxonomic identification was confirmed by Department of Industrial Pharmacy of the Federal University of Santa Maria and registered under the number 225 in the Herbarium of the Industrial Pharmacy Department.

### 2.3. Preparation of the Extract

The leaves were dried for five days in a kiln with controlled temperature (40°C). Aqueous extract was obtained by decoction for 10 minutes in distilled water at 100°C. The resulting extract was then filtered by using a filter paper to remove particles in suspension.* L. divaricata* at 500 mg/kg and 1000 mg/kg were chosen to treat experimental animals based on previous pilot experiment, which demonstrated none toxic effect of the extract. Of particular importance, literature data are not conclusive regarding* L. divaricata* therapeutic dose in animal experiments [27].

### 2.4. Quantification of Compounds by HPLC-DAD

The phenolic compound profiles were determined according to the procedure proposed by Filho et al. [33], with slight modifications. The aqueous extract of* Luehea divaricata* (25 mg/mL) was analysed using a reversed phase carried out under gradient conditions using Phenomenex C_18_ column (4.6 mm × 250 mm) packed with 5 *μ*m diameter particles. Spectral data were recorded from 200 to 700 nm during the whole run. The mobile phase was composed of solvent (A) water : phosphoric acid (99 : 1, v/v) and (B) methanol : water (95 : 5, v/v) and the composition gradient was as follows: 0–5% B in 10 min, 5–20% B in 35 min, 20–50% B in 50 min, and 50–100% B in 70 min. A flow rate of 0.6 mL/min was used, 40 *μ*L of sample was injected, and the wavelengths were 271 nm for gallic acid, 280 nm for catechin and epicatechin, 327 nm for chlorogenic, caffeic, and rosmarinic acids, and 366 nm for luteolin, vitexin, quercetin, and rutin. Samples and mobile phases were filtered through a 0.45 *μ*m membrane filter (Millipore) prior to HPLC injection. Phenolic compounds were identified and quantified by comparing their retention time and UV-visible spectral data to known previously injected standards. Stock solutions of standards references were prepared in the HPLC mobile phase at a concentration range of 0.030–0.450 mg/mL. The chromatography peaks were confirmed by comparing its retention time with those of reference standards and by DAD spectra (200 to 600 nm). Calibration curve for gallic acid is *Y* = 12609*x* + 1187.3 (*r* = 0.9999); catechin is *Y* = 11952*x* + 1308.5 (*r* = 0.9997); epicatechin is *Y* = 11845*x* + 1327.9 (*r* = 0.9999); chlorogenic acid is *Y* = 11695*x* + 1263.7 (*r* = 0.9994); caffeic acid is *Y* = 12704*x* + 1326.8 (*r* = 0.9998); rosmarinic acid is *Y* = 12549*x* + 1243.6 (*r* = 0.9995); vitexin is *Y* = 11895*x* + 1306.7 (*r* = 0.9998); luteolin is *Y* = 13475*x* + 1279.1 (*r* = 0.9999); rutin is *Y* = 12569*x* + 1307.5 (*r* = 0.9997); and quercetin is *Y* = 12409*x* + 1187.3 (*r* = 0.9995). All chromatography operations were carried out at ambient temperature and in triplicate. The limit of detection (LOD) and limit of quantification (LOQ) were calculated based on the standard deviation of the responses and the slope using three independent analytical curves. LOD and LOQ were calculated as 3.3 and 10 *σ*/*S*, respectively, where *σ* is the standard deviation of the response and *S* is the slope of the calibration curve [33].

### 2.5. Animals

All experiments were conducted using male adult Wistar rats (200–250 g) from our own breeding colony. Animals were housed in cages (5 rats per cage) with free access to food and water. They were kept in a 12 h light/12 h dark cycle, with lights on at 7 : 00 a.m., in an air-conditioned room (22 ± 2°C). Commercial diet and tap water were supplied* ad libitum*. Animal care and all experimental procedures were conducted in compliance with the Committee on Care and Use of Experimental Animal Resources (CEUA/UFSM 102/2014). All efforts were made to minimize the number of animals used and their suffering.

### 2.6. 3-NP Induced Neurotoxicity

3-NP was diluted in buffered saline (pH 7.4) and administered intraperitoneally (i.p.) at a dose of 20 mg/kg once a day, for a period of 3 days to induce HD-like symptoms. The 3-NP dose was chosen based in a preliminary study in which were observed biochemistry alterations characteristic of 3-NP neurotoxicity, but with some modifications [13].

### 2.7. Treatment

Twenty-five animals were divided into five groups with five animals each.

Group 1 (control) received pretreatment with distilled water for 7 days, by intragastric gavage.

Group 2 (*L. divaricata*) received daily, during 7 days, the aqueous extract at a concentration of 1000 mg/kg via intragastric gavage.

Group 3 (3-NP) received pretreatment with distilled water for 7 days, by intragastric gavage.

Group 4 (*L. divaricata* + 3-NP) received daily, during 7 days, the aqueous extract at a concentration of 500 mg/kg via intragastric gavage.

Group 5 (*L. divaricata* + 3-NP) received daily, during 7 days, the aqueous extract at a concentration of 1000 mg/kg via intragastric gavage.

On the eighth day, groups 3, 4, and 5 received the administration of 20 mg/kg 3-NP via i.p. [13] for 3 consecutive days, while groups 1 and 2 received only saline (also via i.p.). During the administration of 3-NP, rats continued to receive the aqueous extract by gavage, which results in 10 days of treatment.

All the behavioral parameters were observed on day 10, 3 h after the last 3-NP administration. At the end of the behavioral analyses, rats were euthanized, in a total of 6 h after the last 3-NP administration, the brain was removed, and the cortex and the striatum were dissected. A portion of the cortex and striatum were homogenized (1 : 10) in 10 mM Tris-buffer (pH 7.4) and centrifuged at 2.500 rpm for 12 min. The low-speed supernatant fraction obtained was used for biochemical analyses.

### 2.8. Behavioral Evaluations

#### 2.8.1. Open Field

Animals were individually placed at the center of the open field apparatus (45 cm × 45 cm × 30 cm, divided into 9 squares). Spontaneous ambulation (number of segments crossed with the four paws) and exploratory activity (expressed by the number of rearings on the hind limbs) were recorded for 5 min [34].

#### 2.8.2. Rotarod Task

The integrity of motor system was evaluated using the Rotarod test. Briefly, the Rotarod apparatus consists of a rod 30 cm long and 3 cm in diameter that is subdivided into three compartments by discs from 24 cm in diameter. The rod rotates at a constant speed of 10 rpm. The animals were given a prior training session before the initialization of any therapy to acclimate them to Rotarod apparatus. The latency for first fall and number of falls from the rod were noted. The cut-off time was 120 s [35].

### 2.9. Biochemical Analysis

#### 2.9.1. Estimation of ROS Formation

2′-7′-Dichlorofluorescein (DCF) levels were determined as an index of the reactive species production by the cellular components [36]. Aliquots (20 *μ*L) of homogenate of cortex and striatum were added to a medium containing 2,460 *μ*L Tris–HCl buffer (10 mM, pH 7.4) and 20 *μ*L 2′-7′-dichlorofluorescein diacetate DCFH-DA (0.1 mM). After DCFH-DA addition, the medium was incubated in the dark for 1 h until fluorescence measurement procedure (excitation at 488 nm and emission at 525 nm, and both slit widths used were at 1.5 nm). DCF levels were determined using a standard curve of DCF, and results were corrected by the protein content.

#### 2.9.2. Thiobarbituric Acid Reactive Substances (TBARS) Levels Determination

Lipid peroxidation was determined by measuring thiobarbituric acid reactive substances (TBARS) as described by [37]. An aliquot (200 *μ*L) of homogenate of brain structures (cortex and striatum) was mixed with 500 *μ*L thiobarbituric acid (TBA, 0.6%), 200 *μ*L sodium dodecyl sulphate (SDS, 8.1%), and 500 *μ*L acetic acid (500 mM, pH 3.4) and incubated at 100°C for 1 h. TBARS levels were measured at 532 nm using a standard curve of malondialdehyde (MDA), and the results were reported as nmol MDA/mg protein.

#### 2.9.3. Fluorometric Assay of Reduced (GSH) and Oxidized Glutathione (GSSG)

For measurement of GSH and GSSG levels we used the method previously described by [38]. Briefly, 400 *μ*L of homogenate each of brain structures (cortex and striatum) was mixed to 200 *μ*L trichloroacetic acid (TCA, 13%). Resulting mixtures were centrifuged at 4°C at 13,000 rpm for 10 min. For GSH measurement, 100 *μ*L of the supernatant was diluted in 1,800 *μ*L of phosphate-EDTA buffer (sodium phosphate 100 mM and EDTA 5 mM, pH 8) and 100 *μ*L of O-phthalaldehyde (OPT 1 mg/mL). The mixtures were incubated at room temperature for 15 min and their fluorescent signals were recorded in the RF-5301 PC Shimadzu spectrofluorometer (Kyoto, Japan) at 420 nm of emission and 350 nm of excitation wavelengths.

For measurement of GSSG levels, a 250 *μ*L of the supernatant was incubated at room temperature with 100 *μ*L of N-ethylmaleimide (NEM 0.04 M) for 30 min at room temperature, and after that 140 *μ*L of the mixture was added to 1,760 *μ*L of sodium hydroxide (NaOH, 0.1 N) buffer, following the addition of 100 *μ*L OPT, and incubated for 15 min, using the procedure outlined above for GSH assay. Collectively, data were expressed as a ratio among reduced (GSH) and oxidized (GSSG) glutathione (GSH/GSSG).

#### 2.9.4. Acetylcholinesterase (AChE) Activity

AChE activity was determined according to the method of [38], with some modifications. In brief, we used 875 *μ*L of the reaction mixture, containing potassium phosphate buffer (0.1 M, pH 8), 50 *μ*L 5,5-dithiobis-2-nitrobenzoic acid (DTNB, 10 mM), 25 *μ*L of homogenate of cortex and striatum, and 50 *μ*L acetylthiocholine iodide (9 mM). Change in absorbance was monitored for 2 min at 412 nm.

#### 2.9.5. Protein Determination

The protein content was determined as described previously [39], using bovine serum albumin (BSA) as standard.

### 2.10. Statistical Analysis

Statistical analysis was performed using one-way analysis of variance (ANOVA), followed by multiple comparison test of Newman-Keuls when appropriate. Data are expressed as means ± SEM. Values of *p* < 0.05 were considered significant. Differences between groups of HPLC were assessed by an analysis of variance model and Tukey's test. The level of significance for the analyses was set to *p* < 0.05.

## 3. Results

### 3.1. HPLC Analysis

**Figure 1:**
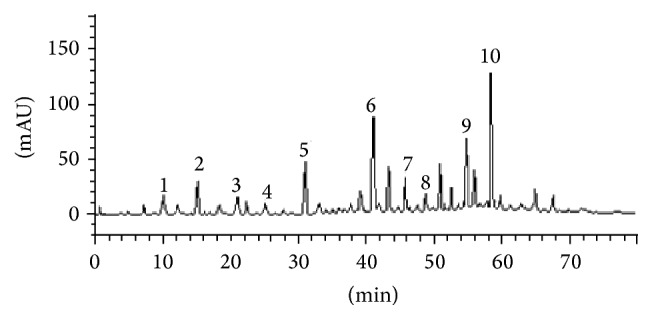
Representative high performance liquid chromatography profile of* Luehea divaricata* aqueous extract. Gallic acid (peak 1), catechin (peak 2), chlorogenic acid (peak 3), caffeic acid (peak 4), epicatechin (peak 5), vitexin (peak 6), rosmarinic acid (peak 7), rutin (peak 8), quercetin (peak 9), and luteolin (peak 10).

HPLC fingerprinting of* Luehea divaricata* aqueous extract revealed the presence of gallic acid (*t*_*R*_ = 9.85 min; peak 1), catechin (*t*_*R*_ = 14.93 min, peak 2), chlorogenic acid (*t*_*R*_ = 21.07 min; peak 3), caffeic acid (*t*_*R*_ = 25.19 min; peak 4), epicatechin (*t*_*R*_ = 31.84 min; peak 5), vitexin (*t*_*R*_ = 41.08 min; peak 6), rosmarinic acid (*t*_*R*_ = 45.98 min; peak 7), rutin (*t*_*R*_ = 48.37 min; peak 8), quercetin (*t*_*R*_ = 54.23 min; peak 9), and luteolin (*t*_*R*_ = 58.11 min; peak 10) (Figure 1 and Table 1).

### 3.2. Behavioral Alterations

**Figure 2:**
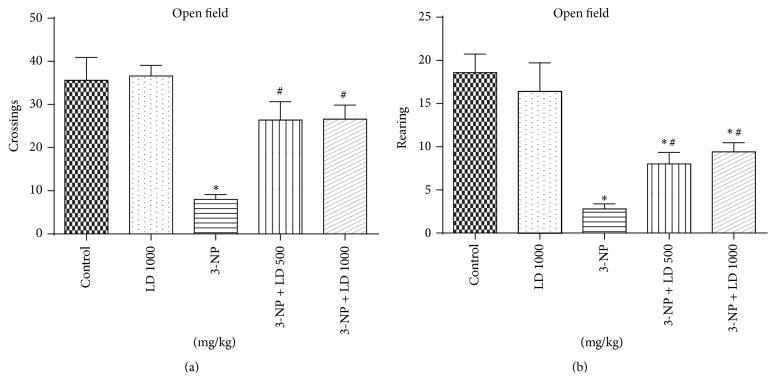
Effects of 3-NP (20 mg/kg, i.p., 3 days) and/or* Luehea divaricata* (LD) (500 and 1000 mg/kg, by gavage, 10 days) on locomotor and exploratory activities. (a) Number of crossings in the open field; (b) number of rearings in the open field. Each bar represents means ± SEM (*n* = 5). *∗* indicates statistic difference from control group and # indicates statistic difference from 3-NP group by one-way ANOVA, followed by Newman Keuls test for* post hoc* comparison (*p* < 0.05).

**Figure 3:**
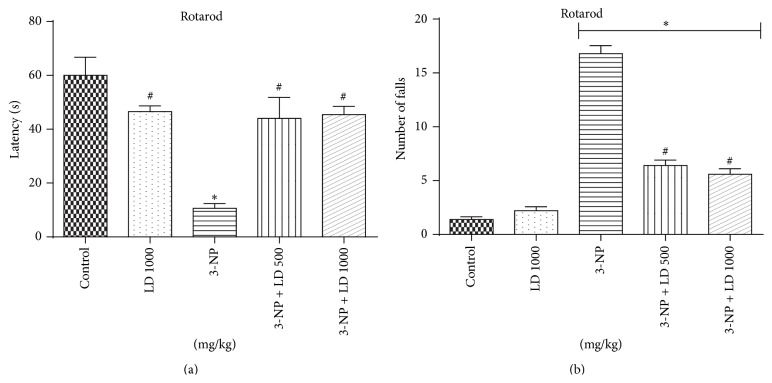
Effects of 3-NP (20 mg/kg, i.p., 3 days) and/or* Luehea divaricata* (LD) (500 and 1000 mg/kg, by gavage, 10 days) on latency to the first fall (a) and number of falls (b) in motor performance of rats in the Rotarod task. Each bar represents means ± SEM (*n* = 5). *∗* indicates statistic difference from control group and # indicates statistic difference from 3-NP group by one-way ANOVA, followed by Newman Keuls* post hoc* test (*p* < 0.05).

Locomotor and exploratory activity in the open field test were significantly decreased following 3-NP administration (Figures 2(a) and 2(b), resp.). Treatment with* L. divaricata* (500 or 1000 mg/kg) partially restored both behavioral parameters to control levels (*p* < 0.05, Figures 2(a) and 2(b)). Additionally, statistical analysis of motor performance in the Rotarod task demonstrated that 3-NP caused a significant reduction of latency to remain on the rotating rod and significantly increased the number of falls off the rod when compared to the control group. Treatment with* L. divaricata* (500 or 1000 mg/kg) was found to completely and significantly attenuate 3-NP-induced changes in Rotarod latency scores and partially restore the animal's ability to remain on the Rotarod (*p* < 0.05, Figures 3(a) and 3(b)). Surprisingly,* L. divaricata* (500 or 1000 mg/kg) treatment was found to significantly decrease the latency to the first fall, when compared to control group (*p* < 0.05, Figure 3(a)).

### 3.3. Biochemical Alterations

**Figure 4:**
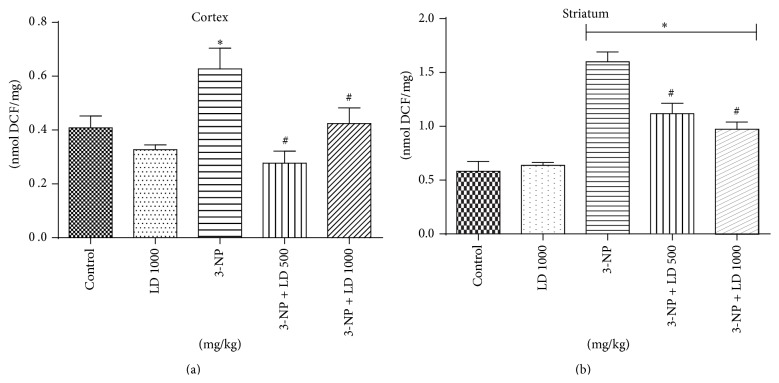
Effects of 3-NP (20 mg/kg, i.p., 3 days) and/or* Luehea divaricata* (LD) (500 and 1000 mg/kg, by gavage, 10 days) on ROS formation in cortex (a) and striatum (b) of treated rats. Data are expressed as nmol DCF/mg. Each bar represents means ± SEM (*n* = 5). *∗* indicates statistic difference from control group and # indicates statistic difference from 3-NP group by one-way ANOVA, followed by Newman Keuls* post hoc* test (*p* < 0.05).

**Figure 5:**
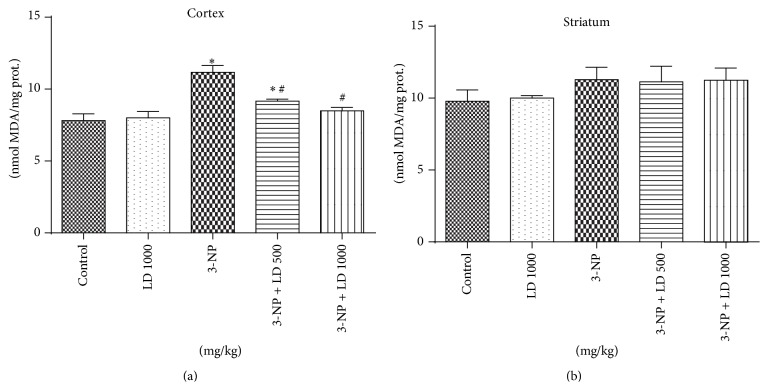
Effects of 3-NP (20 mg/kg, i.p., 3 days) and/or* Luehea divaricata* (LD) (500 and 1000 mg/kg, by gavage, 10 days) on TBARS levels in cortex (a) and striatum (b). Data are expressed as nmol MDA/mg of tissue. Each bar represents means ± SEM (*n* = 5). *∗* indicates statistic difference from control group and # indicates statistic difference from 3-NP group by one-way ANOVA, followed by Newman Keuls* post hoc* test (*p* < 0.05).

Animals treated with 3-NP showed a significant increase (*p* < 0.05) in DCF oxidation, an index of reactive oxygen species (ROS) formation, in both cortex and striatum, when compared with control group (Figures 4(a) and 4(b), resp.).* L. divaricata* treatment completely prevented ROS formation in the cortex (*p* < 0.05, Figure 4(a)), while its effect on striatum was partial (Figure 4(b)). In addition, 3-NP administration significantly increased lipid peroxidation, measured by TBARS production, in the cortex when compared to the control group (*p* < 0.05, Figure 5(a)).* L. divaricata* treatment, at both concentrations, completely prevented the 3-NP-induced increase in TBARS levels in the cortex (*p* < 0.05). Striatal TBARS levels were not modified by 3-NP administration and/or* L. divaricata* treatment (Figure 5(b)).

**Figure 6:**
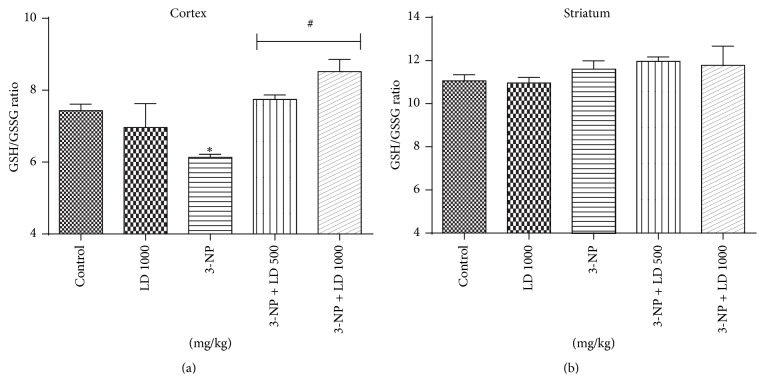
Effects of 3-NP (20 mg/kg, i.p., 3 days) and/or* Luehea divaricata* (LD) (500 and 1000 mg/kg, by gavage, 10 days) on GSH/GSSG ratio in cortex (a) and striatum (b) of treated rats. Data are expressed as nmol GSH/mg of tissue. Each bar represents means ± SEM (*n* = 5). *∗* indicates statistic difference from control group and # indicates statistic difference from 3-NP group by one-way ANOVA, followed by Newman Keuls* post hoc* test (*p* < 0.05).

Administration of 3-NP also caused a marked and significant decrease in the ratio of reduced (GSH) to oxidized (GSSG) glutathione levels in cortex from treated animals (*p* < 0.05, Figure 6(a)). Treatment with* L. divaricata* (500 or 1000 mg/kg) completely restored the GSH/GSSG ratio in the cortex of treated animals (*p* < 0.05, Figure 6(a)). In striatum the ratios in GSH/GSSG levels were not changed by the treatment with 3-NP and/or* L. divaricata* (Figure 6(b)).

**Figure 7:**
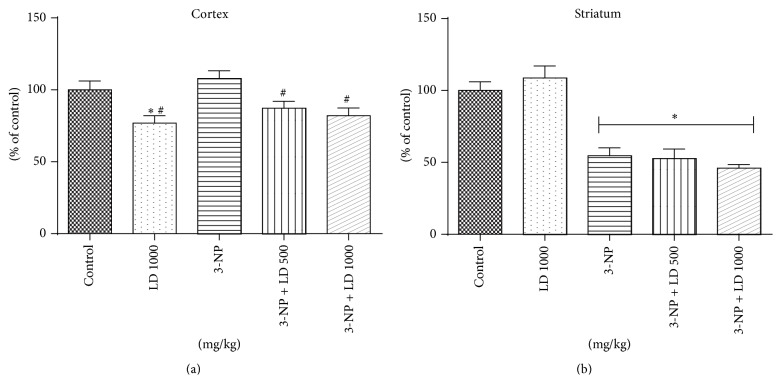
Effects of 3-NP (20 mg/kg, i.p., 3 days) and/or* Luehea divaricata* (LD) (500 and 1000 mg/kg, by gavage, 10 days) on the acetylcholinesterase activity in cortex (a) and striatum (b) of treated rats. Data are expressed as % of control. Each bar represents means ± SEM (*n* = 5). *∗* indicates statistic difference from control group and # indicates statistic difference from 3-NP group by one-way ANOVA, followed by Newman Keuls* post hoc* test (*p* < 0.05).

Administration of* L. divaricata*, either alone or in combination with 3-NP, significantly decreased acetylcholinesterase activity (*p* < 0.05, Figure 7(a)) in the cortex, being the 3-NP without effect* per se*. However, the significant inhibition of activity of acetylcholinesterase activity in the striatum induced by 3-NP administration remained unchanged following* L. divaricata* treatment (500 or 1000 mg/kg; *p* < 0.05, Figure 7(b)).

## 4. Discussion

In the present study we tested the hypothesis that the aqueous extract of* L. divaricata* could prevent behavioral dysfunction and biochemical changes associated with an experimental model of HD induced by 3-NP administration in rats. Our results demonstrate that* L. divaricata* treatment protected against HD-associated behavioral deficits (improved locomotor and Rotarod performance) and attenuated biochemical changes associated with oxidative stress (decreased ROS formation in cortex and striatum, reduced lipid peroxidation, and restored GSH/GSSG ratio in cortex) induced by 3-NP.

Administration of 3-NP in rats for 3 consecutive days caused significant motor dysfunction, characterized by decreased Rotarod and locomotor performance (Figures 2 and 3), suggesting that the effects of 3-NP administration mimic either juvenile onset or later stages of HD-like behaviors in humans [5, 40]. These observations are supported by previous studies reporting that 3-NP administration induces motor system-associated behavioral deficits [3, 41]. Alterations in locomotor behavior may be due to the specific action of 3-NP, those regions of striatum and cortex which control body movement. Additionally, recent studies have indicated that abnormal behavioral symptoms in early HD patients are likely due to either cholinergic interneuron dysfunction in striatal circuits or direct cell loss within the lateral striatum, ventral pallidum, and entopeduncular nucleus [12, 42]. Previous reports have also confirmed 3-NP-induced lesions and oxidative damage in cortex and hippocampus, which may underlie deficits in motor performance [43, 44].

In the present study, pretreatment with* L. divaricata* significantly attenuated behavioral alterations (locomotor function as well as Rotarod performance) following 3-NP administration, suggesting that this compound may have novel therapeutic potential for the treatment of HD and related disorders (Figures 2 and 3). Previous studies support the use of antioxidant therapy to restore behavioral function and oxidative defense levels in 3-NP-treated animals [45, 46]. Using other plant species, a previous study [47] has reported that the root extract of* Withania somnifera*, characterized by high antioxidant content, reverses motor dysfunction caused by 3-NP in rats. Treatment with antioxidants (polyphenols principally) has also been shown to protect* in vivo* against oxidative damage in a model of childhood-onset hydrocephalus in rats and it was found to be effective in improving learning and memory in senescence-accelerated mice including Alzheimer transgenic mice [48]. Thus, considering the presented results, the use of* L. divaricata* aqueous extract could be considered as a therapeutic strategy for the treatment and/or search for new drugs to treat/prevent human HD-like symptoms [13, 49]. However, despite no significant effect on locomotor activity, as assessed by open field test, treatment with* L. divaricata* was found to significantly decrease the latency to first fall in Rotarod test (Figure 3). This result was unexpected and without correlation with the other findings in our study; however, this result pointed out here deserves further attention in future studies with this plant in order to detect possible side effects of extract administration. Moreover, evidence suggests the involvement of oxidative stress in 3-NP neurotoxicity that includes a rapid increase in ROS production in neuronal cells [50] and hydroxyl free radicals, lipid peroxidation, and impaired antioxidant defense in the brain [51]. Accordingly, in this study we found a prooxidant effect of the 3-NP, which caused an increase in ROS production (Figure 4), as measured via DCF oxidation, and in lipid peroxidation (Figure 5), both in cortex and in striatum. These changes were significantly restored by pretreatment with* L. divaricata* extract, suggesting neuroprotective action due to its antioxidant effect. In fact, many studies indicate that the antioxidant activities of aqueous extracts of plants are benefits to the treatment of several diseases by the presence of numerous polyphenols, especially flavonoids [52, 53], which are much more effective than vitamins C and E in protecting cells from free radical damage [54]. In support to this notion, we found a lot of polyphenols in our extract (Figure 1 and Table 1) that could be active in our study, thus preventing against 3-NP-induced oxidative changes, and consequently against 3-NP-induced locomotors impairment.

Alterations in the antioxidant defense system were also observed in this study, as evidenced by a decrease in concentration of GSH/GSSG ratio in the cortex of 3-NP-treated rats (Figure 6(a)). GSH, a nonenzymatic antioxidant, plays an important role in reduction of ROS in brain. So, diminished GSH/GSSG levels have been linked with normal aging and neurodegenerative diseases [41, 55]. Moreover, treatment with* L. divaricata* significantly prevented 3-NP-induced GSH/GSSG consumption. Antioxidants have also been shown to protect the nervous system against variety of toxins [13, 56]. A previously published report [57] demonstrated the efficacy of combined fish oil and quercetin to enhance GSH levels in 3-NP-treated animals.

Finally, we found that aqueous extract* L. divaricata* inhibited acetylcholinesterase activity, which could be due to specific compounds present in the aqueous extract. Previous studies have demonstrated that the compound rutin is an acetylcholinesterase (AChE) inhibitor in human plasma* in vitro* [58] suggesting that* L. divaricata* leaf extract may have anticholinesterase activity* in vivo* [22]. Extracts of* L. divaricata* may therefore be useful in treatments where acetylcholinesterase inhibition is employed, including neurological disorders such as AD. Despite advances in the field, AD remains a devastating neurodegenerative disease with limited therapeutic options. One of the most useful approaches for the treatment of AD is based on the development of AChE inhibitors to attenuate disease-associated deficits of cerebral acetylcholine levels [59]. In addition to its anticholinesterase activity,* L. divaricata* extract may also be useful in AD due to the presence of the compound quercetin, which possesses antioxidant activity, enhances neuronal function, and decreases extracellular *β*-amyloidosis in addition to other beneficial effects on the nervous system including a protective effect on cognitive and emotional function in aged triple-transgenic AD mice [60, 61].

## 5. Conclusion

Our study demonstrates that the aqueous extract of* L. divaricata* is able to prevent oxidative and behavioral changes in an experimental model of HD induced by treatment with 3-NP in rats. These results contribute to the body of knowledge concerning plant extracts and their various components that may be used as novel therapeutic strategies and suggest that this unique plant may be potentially efficacious in the prevention or treatment of neurodegenerative diseases, including HD.

## Figures and Tables

**Table 1:** Composition of *Luehea divaricata* aqueous extract.

Compounds	*Luehea divaricata*		LOD	LOQ
	mg/g	%	*μ*g/mL	*μ*g/mL
Gallic acid	3.51 ± 0.02^a^	0.35	0.025	0.078
Catechin	6.27 ± 0.01^b^	0.62	0.018	0.059
Chlorogenic acid	3.42 ± 0.01^a^	0.34	0.009	0.031
Caffeic acid	1.68 ± 0.03^c^	0.16	0.011	0.037
Epicatechin	8.31 ± 0.01^d^	0.83	0.024	0.071
Vitexin	15.07 ± 0.01^e^	1.50	0.013	0.049
Rosmarinic acid	6.12 ± 0.02^b^	0.61	0.029	0.091
Rutin	1.59 ± 0.01^c^	0.15	0.010	0.034
Quercetin	10.76 ± 0.03^f^	1.07	0.030	0.096
Luteolin	19.45 ± 0.01^g^	1.94	0.007	0.023

Results are expressed as mean ± standard error of mean (SEM) of three determinations. Averages followed by different letters differ by Tukey test at *p* < 0.05.
